# Improving mental wellbeing among families and friends of people with alcohol and drug use issues in Darwin, Australia

**DOI:** 10.1192/j.eurpsy.2024.425

**Published:** 2024-08-27

**Authors:** N. Tari-Keresztes, N. Armstrong, H. Gupta, J. A. Smith, S.-A. Endemann, S. Goding

**Affiliations:** ^1^Rural and Remote Health, Flinders University; ^2^Northern Territory Lived Experience Network, Darwin, Australia

## Abstract

**Introduction:**

Families and friends of individuals with alcohol and other drug use (AOD) issues are highly stigmatised and vulnerable, which often leads to social isolation, decreased quality of life, psychosocial vulnerability, heightened distress, less access to social support, and development of maladaptive coping strategies and own mental health challenges and/or AOD use issues. While peer support for families is commonplace in Australia, in Darwin, psychosocial support activities delivered by peers are very sparse.

**Objectives:**

The NT Lived Experience Network (NTLEN), in allyship with a team of researchers from Flinders University, has secured multiple fundings aimed to develop, implement, and evaluate a peer education and recovery program called Circles of Support (CoS) for families and friends of persons with AOD use issues.

**Methods:**

The suitable evaluation approach was co-designed with live experience representatives from NTLEN and other local key stakeholders. It applied a mixed-method approach, including pre and post-program surveys (n=26) and individual interviews with program participants and the program delivery team (n=11). We also used a co-design approach to develop survey instruments to ensure they were strengths-based and recovery-oriented.

**Results:**

While most participants showed sound stress management skills and understanding of stressors at the program start, about 30% did not think they could handle distress if it got worse and did not have the tools to live the life they wanted. Also, about 25% did not know when to ask for help. Many participants (40%) expressed that they were not hopeful about possible changes in their own family context, such as fewer experiences of stress. By the end of the program, participants reported lower stress levels and higher total empowerment scores. The qualitative interviews highlighted the complexities and challenges participant faced in their journeys. Among them, stigma was considered the most critical, especially among participants from culturally and linguistically diverse backgrounds. In some cases, perceived stigma prevented participants from joining the program. The program was well-received and successful in empowering families and friends and improving their own mental wellbeing. Their key learning and experiences included identifying the stage of their situation, learning to cope with challenges, reducing stress, developing hope, experiencing growth, creating a better and more supportive relationship with their loved ones, and implementing self-care on a regular basis.

**Conclusions:**

Our findings emphasise the critical role of peer support for families and friends in improving their mental health and wellbeing. They also draw attention to improving help-seeking behaviours, which may be influenced by stigma, shame and prioritising the person’s needs.

**Disclosure of Interest:**

None Declared

